# Lactobacilli as Anti-biofilm Strategy in Oral Infectious Diseases: A Mini-Review

**DOI:** 10.3389/fmedt.2021.769172

**Published:** 2021-10-20

**Authors:** Barbara Giordani, Carola Parolin, Beatrice Vitali

**Affiliations:** Department of Pharmacy and Biotechnology, University of Bologna, Bologna, Italy

**Keywords:** lactobacilli, probiotics, postbiotics, paraprobiotics, biofilm, oral diseases

## Abstract

The spread of biofilm-related diseases in developed countries has led to increased mortality rates and high health care costs. A biofilm is a community of microorganisms that is irreversibly attached to a surface, behaving very differently from planktonic cells and providing resistance to antimicrobials and immune response. Oral diseases are an excellent example of infection associated with the formation of highly pathogenic biofilms. It is generally accepted that, when the oral homeostasis is broken, the overgrowth of pathogens is facilitated. Among them, *Porphyromonas gingivalis* and *Aggregatibacter actinomycetemcomitans* are the main etiological agents of periodontitis, while *Streptococcus mutans* is strongly associated with the onset of dental caries. Other microorganisms, such as the fungus *Candida albicans*, may also be present and contribute to the severity of infections. Since the common antibiotic therapies usually fail to completely eradicate biofilm-related oral diseases, alternative approaches are highly required. In this regard, the topical administration of probiotics has recently gained interest in treating oral diseases. Thus, the present mini-review focuses on the possibility of using *Lactobacillus* spp. as probiotics to counteract biofilm-mediated oral infections. Many evidence highlight that *Lactobacillus* living cells can impede the biofilm formation and eradicate mature biofilms of different oral pathogens, by acting through different mechanisms. Even more interestingly, lactobacilli derivatives, namely postbiotics (soluble secreted products) and paraprobiotics (cell structural components) are able to trigger anti-biofilm effects too, suggesting that they can represent a novel and safer alternative to the use of viable cells in the management of biofilm-related oral diseases.

## Introduction

Among virulence factors for pathogens, biofilms are associated with a broad array of topical infections, including periodontal diseases, chronic wounds, and vaginosis (1). The burden of biofilm-related disease contributes to patient morbidity and increased mortality rates, thus representing a major public health issue in developed countries, which adds over $1 billion to US hospitalization costs annually (2, 3). Biofilm is a structured community of microorganisms that irreversibly adhere to an abiotic surface or biological tissue and represents a phenotypically distinct state from the planktonic counterpart, particularly in terms of growth rate and gene expression (1, 4).

Biofilms are morphologically distinguishable by the presence of a self-produced extracellular polymeric matrix that encloses microbial cells, offering great protection against chemical disinfectants, antimicrobials, and human immune response. The host immune system is not only ineffective in fully eradicating mature biofilm, but the presence of antibodies in the surrounding environment can contribute to tissue damage (5). Moreover, sessile microbial biofilm cells are up to 1,000-fold more resistant to antibiotics than planktonic cells, mainly due to the difficulty of high-molecular-weight antibiotics to penetrate the viscous matrix and to reach the deeper layers of biofilm (6, 7). The antimicrobial tolerance can be also ascribed to the presence in the biofilm core of a subpopulation of persistent cells, which *in virtue* of their dormant nonproliferative phenotype are not susceptible to mechanisms of action of common antibiotics (6, 8). Other factors, such as the slow growth rate, the presence of multidrug efflux pumps, and stress response regulation, and the peculiar biofilm microenvironment characterized by differences in pH, pCO_2_, and pO_2_, contribute to reducing the susceptibility of biofilm to antimicrobials (9). Furthermore, the higher cell density in biofilms favors the horizontal gene transfer, which in turn, increases the probability of emergence of strains with resistance or altered virulence profiles (10, 11). Although antimicrobial treatment can alleviate the symptoms of infection by killing the free-floating microorganisms disseminated from the adherent population (12), it usually fails to completely eradicate pathogens in the biofilm. As a result, when antibiotic therapy stops, the persistent cells can revert to their phenotype (13), causing a relapse of the condition and potentially leading to recurrent or chronic infections (5). Given the importance of clearance of mature biofilm for the successful resolution of such infections, alternative strategies to the use of antibiotics are highly desirable. These may include the use of plant-derived compounds, antimicrobial peptides from various sources and probiotics (14). Traditionally, probiotic microorganisms, mainly lactic acid bacteria (LAB) and bifidobacteria, have been orally administered to treat or prevent several gastrointestinal disorders, including diarrhea, gastritis, Crohn's disease, colitis, allergies, food intolerances, and obesity (15). In the last years, there has been an increased interest in the topical administration of probiotic cells to overcome the biofilm-related problem of antibiotic resistance. Probiotics are defined as “Live microorganisms which, when administered in adequate amounts, confer a health benefit to the host” (16). Among LAB bacteria, *Lactobacillus* is the largest genus and comprises the most widely used probiotic species (i.e., *L. rhamnosus, L. acidophilus, L. casei, L. plantarum, L. reuteri, L. delbrueckii*). Lactobacilli are gram-positive, nonsporing, nonrespiring rods, and they are considered as Generally Recognized As Safe (GRAS) (17). In the present mini-review, we explored the possibility of using lactobacilli, intended both as living cells and as derivatives, in the treatment of biofilm-mediated oral infections.

## Biofilm-Mediated Oral Diseases

Oral diseases (i.e., periodontitis, gingivitis, and dental caries) are an excellent example of infection associated with the formation of a highly pathogenic biofilm (18, 19). Periodontitis is one of the most prevalent diseases worldwide and is caused by gram-negative bacteria, such as *Porphyromonas gingivalis* and *Treponema denticola* (20), able to form polymicrobial dental plaque biofilms and to produce virulence factors responsible for an exacerbated inflammatory response, which leads to the destruction of the supporting periodontal tissue and eventually teeth loss (19). Severe forms of periodontitis are also associated with the colonization of subgingival sites by *Aggregatibacter actinomycetemcomitans* and the concomitant reduction of commensal bacteria (21).

The treatment of periodontitis includes hygiene practices, mechanical debridement, and eventually the use of antimicrobials. However, these interventions are not enough to achieve long-term stability, especially in more severe cases, and regular maintenance care based on biofilm control is essential to preserve the equilibrium of the oral microbiome (22). This aspect is also pivotal in the management of dental caries. Members of the *Streptococcus* genus, such as *S. gordonii, S. oralis, S. sanguinis*, and *S. salivarius*, promote the initial colonization on dental pellicle and thus allow for subsequent binding of *S. mutans* (23). *S. mutans* is the main etiological agent of dental caries owing to its ability to produce exopolysaccharides (EPS) such as glucan and to rapidly form a mature biofilm on the tooth surface (22). Besides bacteria, the fungal pathogen *C. albicans* can be present in mixed-species biofilms in dental plaque together with *S. mutans* (24) and is a frequent cause of stomatitis in children, elderly, and immunocompromised patients (25). In oral candidiasis, the switch of *Candida* from budding yeast to filamentous hyphae allows for covalent attachment to the oral mucosal surface, followed by biofilm formation, invasion, and tissue damage.

## Lactobacilli: Probiotics to Counteract Oral Pathogenic Biofilms

The use of probiotics to face up to biofilm-mediated oral diseases has been studied during the last 20 years (26). Examples of lactobacilli exerting anti-biofilm activity against pathogens causing oral diseases are reported in Table 1. The oral cavity is densely colonized by approximately 1,000 bacterial species. The current understanding is that the overgrowth of cariogenic microorganisms in dental caries is driven by a shift in the oral homeostasis toward less diversity (37), favored by acidic production from sugars that create optimal microenvironmental conditions to support the proliferation and biofilm formation of acid-tolerant *S. mutans*. Although in the oral cavity, certain strains of lactobacilli can be cariogenic due to their acidogenic and aciduric features, several studies have reported the therapeutic potential of other beneficial *Lactobacillus* strains (38–40). It has been proposed that lactobacilli may hamper oral pathogens in different ways, even if the exact mechanisms are still poorly understood and could vary among different strains (41). However, lactobacilli can interfere with harmful bacteria and fungi through competition for nutrients, co-aggregation, production of antimicrobials (i.e., bacteriocin, hydrogen peroxide, and organic acids), and modulation of the immune system (42). Moreover, there are few studies showing that lactobacilli are able to integrate into the target biofilm and transiently colonize the oral cavity, thus, competing with pathogens for adhesion sites (43). As an example, Romani et al. demonstrated that *L. reuteri* PTA5289 can be temporarily incorporated in oral microbiota during a 6-weeks intervention, causing a delay in the regrowth of *S. mutants* after full-mouth disinfection with chlorhexidine (44). Some authors reported that commercially available *Lactobacillus* strains (*L. rhamnosus* GG, *L. plantarum* 299v, *L. reuteri* PTA5289 and SD2112, *L. paracasei* DSM16671) reduced biofilm formation of *S. mutans* clinical isolates on both smooth glass surface (27) and saliva-coated hydroxyapatite (28). The persistent presence of a *Lactobacillus* strain potentiates its interference with pathogens. For this reason, Wu et al. evaluated the anti-biofilm activity of *Lactobacillus salivarius*, a species frequently found in human saliva and tooth surface of healthy subjects (45). They observed that the co-cultivation of *S. mutans* with a panel of 64 strains of *L. salivarius* resulted in a reduction of biofilm formation up to 69%, which was higher than that obtained with the reference strain *L. rhamnosus* GG. Moreover, *L. salivarius* reduced the expression of genes (*gtfB, gtfC*, and *gtfD*) encoding for three glucosyltransferase (Gtfs) involved in the synthesis of exopolysaccharide matrix, and consequently crucial for *S. mutans* biofilm formation (29). Similarly, Lee at al. demonstrated that *L. rhamnosus* GG exerted an anti-biofilm activity by decreasing the expression of *gtfs* in *S. mutans* but it was not able to integrate into the oral biofilm model. Contrariwise, *L. acidophilus* ATCC4356 and *L. reuteri* ATCC334 integrated into *S. mutants* biofilm without affecting glucan production (30). *S. mutans* produces proteins anchored in the cell walls to facilitate binding to *C. albicans*, which in turn, supports streptococcal colonization and caries progression of the formed biofilm (46, 47). Krzyściak et al. showed that *L. salivarius* (HM6 Paradens) not only reduced the biomass of mono-species biofilms of *S. mutans* and *C. albicans* clinical strains, but also the multispecies biofilm. Interestingly, SEM images revealed that the addiction to viable probiotics inhibited the formation of hyphae and germ tubes in *C. albicans*, consequently hindering the fungal pathogenesis (32). Other authors supported this observation. In particular, three *Lactobacillus* strains isolated from the saliva of caries-free subjects were able to reduce biofilm development when co-incubated with two oral strains of *C. albicans* (35). The anti-biofilm activity seemed to be related to the downregulation of *Candida* biofilm-specific genes, including *HWP1* (a glycoprotein located in the hyphal surface), *ALS3* (a protein similar to alpha-agglutinin that is essential for the adhesion of *Candida*), and *CPH1* (a regulator of morphogenesis implicated in the maintenance of cell wall organization, pseudohyphal formation in response to oxidative stress, and biofilm development) (39, 40). Viela et al. demonstrated that *L. acidophilus* ATCC4356, a strain widely employed as food supplements, inhibited the formation of *C. albicans* ATCC18804 biofilm (57%) and filamentation *in vitro*, with the best results achieved after 24 h of incubation; the reduction in the number of fungal hyphae produced after probiotic treatment was also observed in *Galleria mellonella* infection model (48). James et al. have found that a multistrain probiotic combination (*Lactobacillus helveticus* CBS N116411, *L. plantarum* SD57870, *S. salivarius* DSM14685) was effective at both preventing the formation of (>67%) and removing preformed (>63%) *C. albicans* biofilms (36). Moreover, the combination of two lactobacilli supernatant significantly reduced the expression of several *C. albicans* genes involved in the yeast–hyphae transition, such as *ALS3, EFG1* (hyphae-specific gene activator), *SAP5* (secreted protease), and *HWP1*, in agreement with the results of Rossoni et al. (35).

**Table 1:** Probiotic *Lactobacillus* spp. exhibiting *in vitro* anti-biofilm activity against pathogens involved in oral biofilm-related diseases.

**Target biofilm**	***Lactobacillus* species**	**References**
*S. mutans*	*L. casei*, *L. paracasei*, *L. plantarum*, *L. reuteri*, *L. rhamnosus*, *L. salivarius*	(27–31)
Multispecies (*S. mutans* and *C. albicans*)	*L. salivarius*	(32)
Multispecies (*P. gingivalis, S. oralis, S. gordonii*)	*L. acidophilus*, *L. rhamnosus*, *L. reuteri*	(33)
*A. actinomycetemcomitans*	*L. acidophilus*, *L. casei*, *L. fermentum*, *L. johnsonii*, *L. plantarum*, *L. sake*, *L. paracasei*	(34)
*C. albicans*	*L. fermentum*, *L. helveticus* *L. paracasei*, *L. plantarum*, *L. rhamnosus*	(35, 36)

Regarding periodontal disease therapy, in a study carried out by Jaffar et al., different species of lactobacilli were sought for their ability to eradicate preformed biofilm of three strains of *A. actinomycetemcomitans*, with promising results. In particular, biofilms of *A. actinomycetemcomitans* Y4 (serotype b) and *A. actinomycetemcomitans* OMZ 534 (serotype e) were effectively dispersed after the addiction of lactobacilli, with reduction rates up to 90% and, interestingly, the authors identified three probiotic enzymes, namely protease, lipase, and amylase, that may be responsible for the anti-biofilm activity (34).

## Beyond Probiotics: *Lactobacillus* Derivatives

Beneficial outcomes of lactobacilli can be obtained not only by living cells, but their byproducts (cell components or metabolites) may be also able to trigger probiotic effects (49). The terms “postbiotics” and “paraprobiotics” have emerged recently and have gained increasing interest since they can exert a wide range of positive effects on the host, such as immunomodulatory, antitumor, and antimicrobial activities, as well as preservation of intestinal barrier (50). Postbiotics are defined as soluble products or metabolites secreted by probiotics capable of providing physiological benefits through direct or indirect mechanisms. They include metabolic byproducts of live probiotic cells, such as cell-free supernatant (CFS), secreted proteins, bacteriocins, organic acids, and secreted biosurfactants (BS) (51). The term paraprobiotics is used to indicate nonviable probiotics (inactivated or dead intact cells), or their cell structural components that can be recovered after cell rupture. The latter comprise peptidoglycans, teichoic acid, cell wall polysaccharides, surface proteins, and cell wall-bound BS (52). Concerns about the administration of living probiotics have been described in experimental models, clinical trials, and case reports (50, 53). Postbiotics/paraprobiotics display prolonged shelf life and good stability (54) and, in this regard, can be potentially employed in oral therapy as a safer alternative to the use of viable cells. Lactobacilli postbiotics/paraprobiotics with anti-biofilm properties toward oral pathogens are listed in Table 2 and briefly described in the following sections.

**Table 2:** Postbiotics/paraprobiotics from *Lactobacillus* spp. exhibiting *in vitro* anti-biofilm activity against pathogens involved in oral biofilm-related diseases.

**Postbiotics/paraprobiotics**	**Dosage**	***Lactobacillus* species**	**Target biofilm**	**References**
Lipoteichoic acid	10-30 μg/mL	*L. plantarum*	*S. mutans*	(55)
	30-50 μg/mL	*L. plantarum*	Multispecies (*A. naeslundii, L. salivarius, S. mutans* and *E. faecalis*)	(56)
Polysaccharide	Na	*L. reuteri*	*S. mutans*	(57)
Biosurfactant	2.51–0 mg/mL	*L. acidophilus*	*S. mutans*	(58–61)
	2.5 mg/mL	*L. casei*		
	1–10 mg/mL	*L. paracasei*		
	1–10 mg/mL	*L. reuteri*		
	1–10 mg/mL	*L. Rhamnosus*		
	10 mg/mL	*L. acidophilus*, *L. paracasei*, *L. reuteri*, *L. rhamnosus*	*S. oralis*	(58)
Cell-free supernatant		*L. casei*, *L. fermentum*, *L. johnsonii*, *L. kefiranofaciens*, *L. paracasei*, *L. plantarum*, *L. rhamnosus*	*S. mutans*	(31, 62–64)
		*L. brevis*	*S. salivarius*	(65)
		*L. johnsonii*, *L. kefiranofaciens*, *L. plantarum*, *L. rhamnosus*	*S. sobrinis*	(63)
		*L. plantarum*	Multispecies (*S. mutans* and *C. albicans*)	(64)
		*L. reuteri*, *L. rhamnosus*	Multispecies (*P. gingivalis, S. oralis, S. gordonii*)	(33)
		*L. acidophilus*, *L. casei*, *L. fermentum*, *L. johnsonii*, *L. rhamnosus*, *L. plantarum*, *L. sake*, *L. paracasei*	*A. actinomycetemcomitans*	(34, 66)
		*L. casei*, *L. fermentum*, *L. paracasei*, *L. plantarum*, *L. rhamnosus*	*C. albicans*	(35, 64, 67)

*Na, not available*.

### Lipoteichoic Acid

Teichoic acids are the second main constituent of cell walls of lactobacilli and can be covalently linked to peptidoglycan as wall teichoic acid or anchored to the cytoplasmic membrane as lipoteichoic acid (LTA) (68). *L. plantarum* LTA is known to possess anti-biofilm activity against *Staphylococcus aureus* (69) and *Enterococcus faecalis* (70). Ahn et al. demonstrated that it was also able to prevent the biofilm formation of *S. mutans* on polystyrene plates, hydroxyapatite disk, and dentine slices without affecting streptococcal growth, but interfering with sucrose decomposition essential for the production of EPS (55). Furthermore, *L. plantarum* LTA disrupted preformed oral multispecies biofilm (*Actinomyces naeslundii* ATCC12104, *L. salivarius* ATCC11741, *S. mutans* KCTC3065, and *E. faecalis* ATCC29212) in a dose-dependent manner and potentiated the effectiveness of common intracanal medicaments (i.e., calcium hydroxide and chlorhexidine digluconate) (56).

### Polysaccharides

The most studied polysaccharides are EPS. Since their production is highly strain-specific and depends on several variables (i.e., medium, pH, age of culture), EPS greatly varies in the degree of branching and monosaccharide composition. EPS mediates the interactions between bacteria and the environment and protects against hostile conditions and immune response. On the other hand, *Lactobacillus*-derived EPS revealed antimicrobial and anti-biofilm activities toward a broad range of pathogens, including *E. faecalis, S. aureus, Escherichia coli*, and *Pseudomonas aeruginosa* (71). Moreover, *L. reuteri* BM53-1 was found to produce a short extracellular polysaccharide with a molecular weight of about 30 kDa that impeded the production of sticky β-glucans by *S. mutans* and consequently the biofilm formation. In particular, the authors suggested that it acts by lowering the expression of *gtfD* that is necessary to give sickness to insoluble glucans produced in large amounts during the initial attachment of *S. mutans* on the surface (57).

### Biosurfactants

Biosurfactants are secondary metabolites consisting of complex polymeric mixtures (i.e., glycolipids, lipopeptides) that can be secreted extracellularly or bounded to the cell wall. *In virtue* of their amphiphilic nature, BS exhibits emulsification properties and may assist in the dispersal of preformed biofilms or preventing the onset of pathogenic biofilms (52). Some studies highlighted that BS may be used as an alternative to antibiotics to decrease the chance of dental caries by acting as anti-biofilm agents toward *S. mutans*. For instance, the anti-adhesive activity of *L. paracasei* BS against several bacteria, including *S. mutans* and *S. oralis*, was reported by Gudiña et al. (72). Furthermore, excreted BS purified from cultures of *L. reuteri* DSM17938, *L. acidophilus* DDS-1, *L. rhamnosus* ATCC53103, and *L. paracasei* B21060 reduced the adhesion and biofilm formation on the titanium surface of *S. mutans* and *S. oralis* in a dose-dependent manner (58). Evidence shows that the anti-biofilm behavior of both excreted and cell-bound *Lactobacillus* BS can be ascribed to the downregulation of Gtfs genes, mainly *gtfB* and *gtfC*, as previously described for living probiotic cells (59–61). In addition, the expression of *ftf* , a proadhesive gene encoding for *S. mutans* fructosyltransferase, resulted significantly reduced in the presence of BS produced by *L. acidophilus* DSM20079 (59), *L. casei* ATCC39392 (60), and *L. rhamnosus* ATCC7469 (61).

### Cell-Free Supernatant

Cell-free supernatant of lactobacilli is a consortium of low (i.e., organic acids, hydrogen peroxide, and reuterin) and high molecular weight (i.e., bacteriocins and bacteriocin-like polypeptides) metabolites (52). Several findings suggested that *Lactobacillus*-derived CFS acts as bio-liquid-detergent reducing the adhesion and biofilm formation of pathogens to abiotic and biotic surfaces. The formation of biofilms of multidrug resistant superbugs, namely *P. aeruginosa* and *S. aureus*, were successfully mitigated by CFS recovered from different *Lactobacillus* species, including *L. casei, L. fermentum, L. gasseri, L. plantarum*, and *L. salivarius* (73–77). Several authors investigated the effects of CFS deriving from *Lactobacillus* spp. commonly used as dietary products on the development of dental caries. As an example, *Lactobacillus brevis* FF2, isolated from fermented oil, exerted anti-biofilm activity against *S. salivarius*. Clinically isolates belonging to *L. paracasei* and *L. fermentum* species significantly reduced the number of cariogenic *S. mutans* UA159 cells in biofilm grown on hydroxyapatite (62), while Lin et al. found that CFS from five lactobacilli inhibited the biofilm formation of the same pathogen on the glass surface. The acidic environment produced by CFS seemed to be important to elicit the anti-biofilm effects since the activity was lost for three strains out of five after adjusting the pH of CFS to 6.5 (31). Moreover, Jeong et al. observed that *Lactobacillus*-derived CFS caused a variation in streptococcal expression of three classes of biofilm-formation associated genes, namely those involved in carbohydrate metabolism, adhesion, and biofilm structure (63). Srivastava et al. reported that *L. plantarum* 108 CFS inhibited the formation of *S. mutans* and *C. albicans* mixed biofilm by 85%, and also reduced preformed biofilm by 33%. As observed for living cells, probiotic CFS downregulated the expression of *S. mutans* genes associated with Gtfs activity (*gtfB, gtfC*, and *gtfD*) and *C. albicans* genes involved in adhesion (*ALS3* and *ALS1*) and hyphae formation (*HWP1*) (64). Furthermore, *Lactobacillus*-derived CFS altered biofilm formation and the transcription of virulence-associated genes of periodontal pathogens. The anti-biofilm activity exerted by *L. rhamnosus* (Lr32 and HN001) and *L. acidophilus* (LA5 and NCFM) CFS was associated with a diminished expression of two exotoxins of *A. actinomycetemcomitans* (leukotoxin and CDT) and with the downregulation of *katA*, encoding a catalase, which promote *Aggregatibacter* survival under oxidative stress (66). *L. acidophilus* LA5 CFS also reduced the abundance of *P. gingivalis* in multispecies biofilm along with *S. oralis* and *S. gordonii*. In this case, a panel of *P. gingivalis* genes resulted downregulated, including *fimA* (encoding the main fimbriae), *MFA1* (involved in auto-aggregation), *kgp* and *rgp* (involved in EPS accumulation), and *Lux6* (pivotal in quorum sensing) (33).

## Conclusions

Therapies for periodontal diseases are costly and may not be sufficiently effective. Findings explored in the present mini-review highlighted that lactobacilli can behave as anti-biofilm agents against a variety of microorganisms responsible for oral diseases, although the activity strongly varies among different *Lactobacillus* strains. Notably, not only living cells but also *Lactobacillus* derivatives may exert antipathogenic effects. Thus, the employment of postbiotics, paraprobiotics, or a mixture of both can represent a novel bio-therapeutic approach to face biofilm-related oral diseases, favoring the formulation stability and safety. It is also a step forward in the search for alternative solutions to the use of antibiotics, in the perspective of containing the spread of antibiotic resistance.

## Author Contributions

All authors listed have made a substantial, direct and intellectual contribution to the work, and approved it for publication.

## Funding

This study was supported by the Italian Ministry for University and Research.

## Conflict of Interest

The authors declare that the research was conducted in the absence of any commercial or financial relationships that could be construed as a potential conflict of interest.

## Publisher's Note

All claims expressed in this article are solely those of the authors and do not necessarily represent those of their affiliated organizations, or those of the publisher, the editors and the reviewers. Any product that may be evaluated in this article, or claim that may be made by its manufacturer, is not guaranteed or endorsed by the publisher.
